# History, Developments and Trends in the Heat Treatment of Steel

**DOI:** 10.3390/ma13184003

**Published:** 2020-09-09

**Authors:** Peter Jurči

**Affiliations:** Faculty of Materials Science and Technology in Trnava, Institute of Materials Science, Slovak University of Technology in Bratislava, 917 24 Trnava, Slovakia; p.jurci@seznam.cz

**Keywords:** grade 92 steel weldment, post-welding heat treatment, tensile straining, hydrogen embrittlement, ledeburitic tool steels, carburizing, rare-earth element pre-implantation, sub-zero treatments, microstructure, hardness, toughness, microstructure, fractography

## Abstract

Ferrous alloys (steels and cast irons) and their heat treatment have attracted a great amount of basic and applied research due to their decisive importance in modern industrial branches such as the automotive, transport and other industries. Heat treatment is always required for these materials, in order to achieve the desired levels of strength, hardness, toughness and ductility. Over the past decades, many advanced heat- and surface-treatment techniques have been developed such as heat treatment in protective atmospheres or in vacuum, sub-zero treatment, laser/electron beam surface hardening and alloying, low-pressure carburizing and nitriding, physical vapour deposition and many others. This diversity of treatment techniques used in industrial applications has spurred a great extent of research efforts focused on the optimized and/or tailored design of processes in order to promote the best possible utilization of material properties. This special journal issue contains a collection of original research articles on not only advanced heat-treatment techniques—carburizing and sub-zero treatments—but also on the microstructure–property relationships in different ferrous alloys.

Advanced heat- and surface-treatment techniques play a dominant role in material processing in modern industry because of the high level of protection against unwanted surface defects such as oxidation, decarburization, and too-high shape and dimensional distortion, thus reducing the final operations that are often necessary in order to correct these phenomena. Among these processes, vacuum heat treatment became of great importance in the processing of tool steels and other high-alloyed materials, owing to the fact that it produces clean surfaces after heat treatment and that the vacuum furnaces can be directly incorporated into the manufacturing lines [1]. Thermo-chemical processes such as carburizing or nitriding often use atmospheres with hazardous environmental and health impacts such as endo-gases or ammonia. Advanced thermo-chemical processing techniques such as low-pressure carburizing or nitriding, on the other hand, produce much lower amounts of environmentally dangerous substances, produce clean surfaces on tools and components, and reduce their distortion [2,3,4]. Moreover, these processes manifest much better efficiency, i.e., they enable the production of surface regions with greater thickness and better uniformity, with substantially shorter processing times.

In production of hard ceramic layers, processes such as chemical vapour deposition (CVD), which often produce poisonous hydrogen chloride as a by-product, were replaced by much more environmentally friendly physical vapour deposition processes. These treatment techniques, in addition, enable the design and manufacture of tailored-to-customer thin-film architecture, composition and thickness [5].

Research efforts have also been focused on the design and utilization of the high-power density treatment of metallic surfaces. Among the techniques, laser- and electron-beam surface hardening, remelting and alloying became of the greatest importance in research, development and industry. This is owing to the possibility to set up the processing parameters exactly in order to obtain the desired thickness, microstructure and related properties of treated surfaces [6].

This Special Issue contains five original, full-length articles on the effects of surface quality on the mechanical properties of hard steels, on sub-zero treatments and their impact on microstructure and mechanical properties, and on the advanced carburizing technique. 

Jurči [7] reported on the worsening of the bulk toughness of hard tool steels, which results not only from increased surface roughness but also from the application of thermo-chemical treatments such as carburizing, nitriding or boronizing. On the other hand, almost no effect of physical vapour deposited (PVD) hard coatings on toughness is reported. The material toughness is exactly quantified by means of measurements of flexural strength and by the estimation of the plastic work of fracture. Toughness measurements are complemented by a thorough analysis of the fractured surfaces.

In the second paper [8], Razavykia, Delprete and Baldissera provide a comprehensive review on the cryogenic treatment of metallic materials. They discuss the improvement of material properties and durability by means of microstructural alteration comprising phase transfer, particle size adjustment, and distribution. These effects are almost permanent and irreversible. However, while improvements in the properties of materials after cryogenic treatment are discussed by the majority of reported studies, the correlation between microstructural alteration and the mechanical properties is unclear to date. At the end of the paper, the development and the trends for future research in this field are outlined and discussed.

The third paper [9] deals with the carburizing of 20Cr2Ni4A alloy steel, which was pre-implanted with either lanthanum or yttrium. The obtained results showed that rare-earth elements promoted the formation of add-on dislocations in the surface areas, which increased the carbon diffusion coefficient and thereby contributed to better carbon distribution in the carburized layers. This was reflected in the improved hardness of the low-pressure carburized components. At the end of the paper, it is stated that yttrium acts better in terms of obtaining increased surface hardness as compared to the lanthanum ion. 

Kusý et al. [10] treated the Vanadis 6 steel at the cryogenic temperature of −75 °C, for different durations. They arrived at the principal findings that this kind of treatment reduces the retained austenite amount to one third as compared with that in the same steel after room-temperature quenching. Moreover, treatment at −75 °C produces a great number of extra small globular carbides in the material microstructure. This improves the prior-to-tempering hardness. However, sub-zero treatment at −75 °C leads to the complete loss of the secondary hardness peak, and the as-tempered bulk hardness manifests clear lowering. The material toughness was slightly deteriorated when the steel was low-temperature tempered, but an improvement was recorded after high-temperature tempering. The obtained results were compared with those after sub-zero treatments at −140, −196 or −269 °C.

In the fifth paper [11], the effects of the electrolytic hydrogen charging of T92 steel weldments on their room-temperature tensile properties were investigated and discussed. The weldments were differently heat treated after the welding procedure—either tempered below the transformation A_1_ temperature or normalized (i.e., austenitized above the Ac_3_ critical transformation temperature and subsequently air cooled) and tempered. The obtained results indicated higher hydrogen embrittlement susceptibility for the normalized-and-tempered weldments, compared to the tempered-only ones. The obtained findings were correlated with performed microstructural and fractographic observations.

All the published articles were reviewed by recognized experts in the appropriate fields through a single-blind peer-review process. As Guest Editor, I would like to acknowledge all of the authors for their valuable contributions to the Special Issue. I would also like to thank the reviewers for their comments and suggestions that greatly improved the quality of the papers. Finally, I would like to thank the Section Managing Editor, Ms. Ariel Zhou, for her kind assistance in the preparation of the Special Issue of the journal.
